# Seizure as the Main Manifestation of Nonalcoholic Wernicke’s Encephalopathy but Without Cortical Involvement: A Case Report

**DOI:** 10.7759/cureus.28866

**Published:** 2022-09-06

**Authors:** Mohammad Abu-Abaa

**Affiliations:** 1 Department of Internal Medicine, Capital Health Regional Medical Center, Trenton, USA

**Keywords:** wernicke’s encephalopathy, nonalcoholic wernicke’s encephalopathy, horizontal nystagmus, cortical, seizure activity

## Abstract

Wernicke’s encephalopathy (WE) is an underdiagnosed entity. A seizure can be the main manifestation of WE even without cortical involvement. This is a case report of a 45-year-old female patient with a past medical history of depression and poor oral intake who presented with a single episode of unwitnessed seizure and three days of unsteady gait and vertigo. She then had two episodes of seizure, focal and then generalized tonic. Her physical examination was remarkable for lethargy and bilateral gaze-induced horizontal nystagmus with a rotational component and change in direction. Magnetic resonance imaging (MRI) of the brain with contrast showed non-enhancing bilateral symmetrical fluid-attenuated inversion recovery (FLAIR) hyperintensities in the medial thalami and tectum. Vitamin B1 level was found to be low. Lumbar puncture (LP) was unyielding. She was loaded with high-dose thiamine replacement. After a few days, a neurological examination revealed improvement with unilateral nystagmus with less lethargy. The valproate that was started initially was eventually discontinued during follow-up after the resolution of neurological deficits. Interestingly, baseline echocardiography showed heart failure with reduced ejection fraction at 40% with clinical euvolemia. It was believed to be secondary to beriberi.

## Introduction

Wernicke’s encephalopathy (WE) is a potentially reversible acute neurological disease but, if missed, can result in significant neurological sequelae. Korsakoff syndrome is the late irreversible stage of untreated WE where memory impairment becomes evident with confabulations. It results from thiamine deficiency and is classically characterized by the triad of ataxia, confusion, and ophthalmoplegia. The diagnosis remains largely clinical. Magnetic resonance imaging (MRI) is helpful to make the diagnosis but is not required. Vitamin B1 level is limited in specificity but can aid to establish the diagnosis. WE is typically seen among alcoholics. However, clinicians should remain vigilant about the possibility of nonalcoholic WE. There are several predisposing factors to nonalcoholic WE. Treatment with thiamine supplementation usually results in the recovery of most of the clinical manifestations, although at a different pace. We present a patient with nonalcoholic WE who presented with focal seizure with secondary generalization. Interestingly, no cortical lesion is observed. We believe that this is a good case to report as a seizure as the initial presentation of WE was reported only in 14 cases in the English literature. This case also shows that early thiamine replacement can result in significant neurological improvement [1]. In this case report, we aim to review what the English literature has to answer the questions about the differences between alcoholic and nonalcoholic WE, the risk factors of nonalcoholic WE, the typical and atypical MRI findings, and the prevalence of seizure and cortical lesion in WE.

## Case presentation

A 45-year-old female patient with a past medical history of depression on selective serotonin inhibitors complicated by poor oral intake presented for evaluation of a 90-second episode of loss of consciousness on the same day of presentation with the prior three days of unsteady gait and vertigo. Otherwise, her history was unremarkable. She denied a history of seizures and alcohol intake. While in the emergency department (ED), she was noticed to have a focal seizure lasting for a few minutes in the form of focal twitching of the right side of the face and neck while maintaining consciousness. Her vital signs were within normal ranges. Her physical examination was remarkable for lethargy and bilateral gaze-induced horizontal nystagmus with a rotational component and change in direction. Gait examination showed mild gait instability. Otherwise, the physical examination including reflexes, motor and sensory function, and coordination was unremarkable. Basic laboratory tests were remarkable for normochromic normocytic anemia at 11 g/dL with a mean corpuscular volume (MCV) of 85 fL.

**Figure 1:**
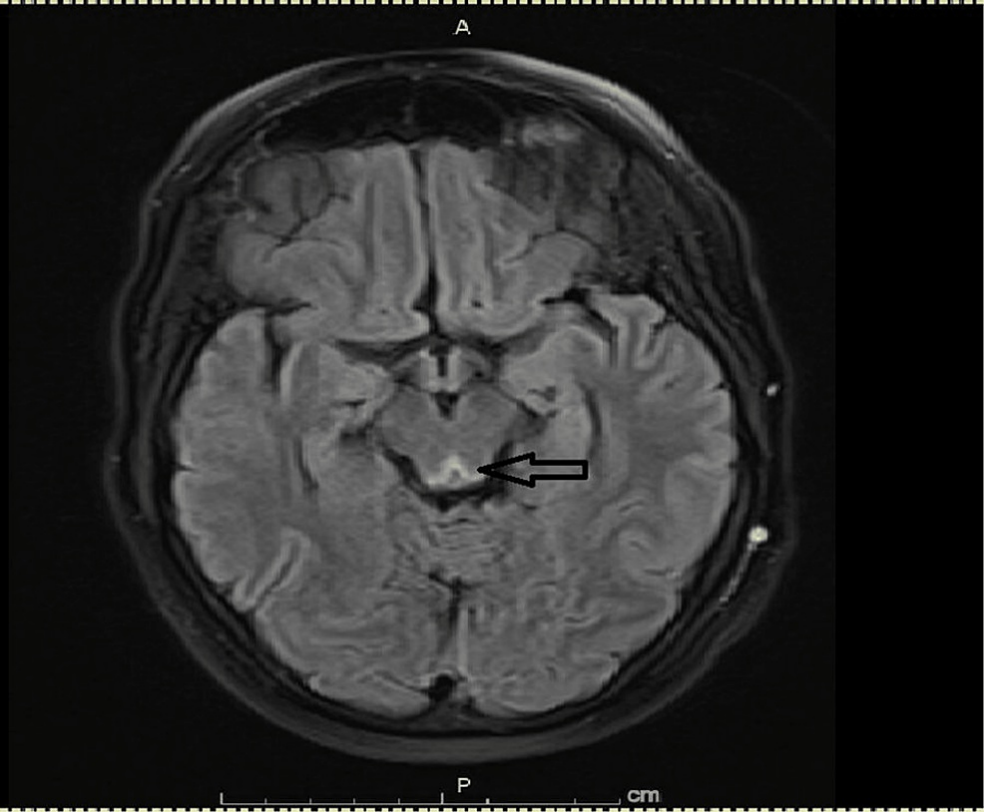
FLAIR MRI tectal hyperintensity FLAIR MRI of the brain shows hyperintensity over the tectum (arrow).

**Figure 2:**
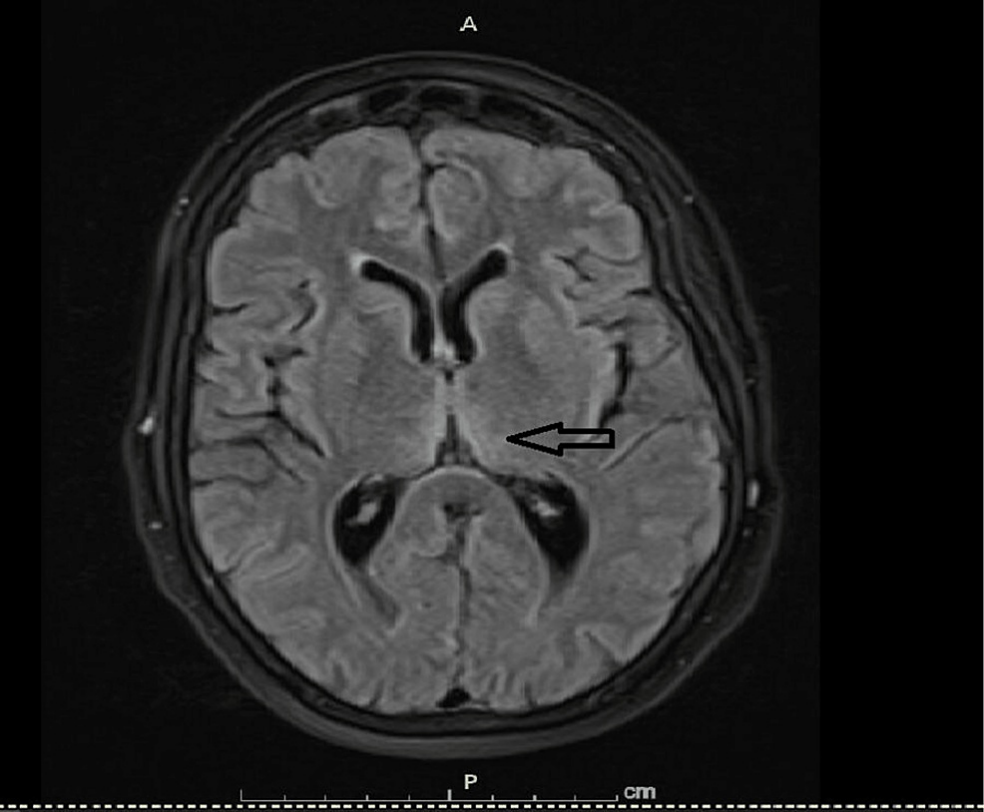
FLAIR MRI medial thalamic hyperintensities FLAIR MRI of the brain shows vague bilateral hyperintensities in both medial thalami (arrow).

The patient was loaded with intravenous valproate. MRI of the brain with contrast showed non-enhancing bilateral symmetrical fluid-attenuated inversion recovery (FLAIR) hyperintensities in the medial thalami and tectum (Figure 1 and Figure 2). No other abnormalities were seen on imaging. Continuous video electroencephalogram (EEG) monitoring showed no epileptiform discharges. A lumbar puncture (LP) was pursued, but cerebrospinal fluid (CSF) analysis was unyielding. It showed no pleocytosis and normal protein and glucose. CSF culture and meningitis/encephalitis panel were unreactive. Venereal Disease Research Laboratory (VDRL) test, West Nile virus (WNV), and paraneoplastic encephalitis serology were negative.

She had an episode of generalized tonic seizure while on the medical floor and on valproate. Constellation of MRI findings with nystagmus was suggestive of Wernicke’s encephalopathy. Although it is not required for diagnosis, vitamin B1 level was noted to be low at 33 nmol/L (normal range: 66-200 nmol/L). She was started on three days of 500 mg intravenous thiamine supplementation followed by five days of 250 mg intravenous thiamine. Her neurological examination revealed improvement with unilateral nystagmus. Afterward, daily oral thiamine supplementation was pursued. Valproate was eventually discontinued four months later during follow-up after the resolution of neurological deficits. However, she refused a follow-up MRI due to claustrophobia.

Interestingly, baseline echocardiography showed heart failure with a reduced ejection fraction at 40%. No structural abnormalities were seen. She was clinically euvolemic. Guideline-directed medical therapy (GDMT) was gradually introduced. It was believed to be secondary to beriberi. Follow-up echocardiography five months later showed improvement of her ejection fracture to above 55%.

## Discussion

Epileptic seizures are rare in nonalcoholic WE. It may or may not be associated with cortical lesions. A recent literature review reported 14 cases of nonalcoholic Wernicke’s encephalopathy with seizure. Only six of those displayed cortical involvement [1]. Among those patients, only three had seizures as the initial presentation. Thus, we believe that this case is worthy of publishing due to the rarity of this occurrence. No exact frequency of seizure was found in the English literature among those with WE. Another retrospective study found that the frontal and parietal lobes, especially around the central sulcus, are the most susceptible areas [2]. Cortical involvement is usually seen as a sign of irreversible damage and poor prognosis [3].

A multicenter observational study of 468 patients with WE found that those with alcoholic WE as compared to nonalcoholic WE are more likely to present with cerebellar signs and less likely to have ocular signs. Those with nonalcoholic WE tend to present with altered mental status, and hence, diagnosis may also be delayed, and the prognosis is worse [4]. These findings were also redemonstrated in another smaller retrospective study [5]. Also, atypical sites of involvement on brain MRI are more common among those with nonalcoholic WE [6]. Mammillary body atrophy is more common among alcoholics, while nonalcoholics have only signal intensity changes on MRI [3]. The classical triad of confusion, ataxia, and ophthalmoplegia is more common in alcoholic WE than in nonalcoholic WE [7]. Gadolinium enhancement was seen more commonly in alcoholic WE than in nonalcoholic WE. Also, cranial nerve nucleus involvement was seen more commonly in nonalcoholic WE than in alcoholic WE. Similar to cortical involvement, the presence of lesions of the caudate nuclei, frequently observed in patients in a comatose state, is taken as a sign of poor prognosis [8].

It is important to recognize the precipitating situation for nonalcoholic WE. The risk factors of nonalcoholic WE include hyperemesis gravidarum, malnutrition, chronic kidney disease, thyrotoxicosis, anorexia nervosa, organ transplantation, and total parenteral nutrition [9]. Other risk factors also include magnesium deficiency, malignancies, intensive care unit admission, bariatric surgeries, and a defective soy-based formula described in children and adolescents with WE. Genetic defects in the *SLC19A3* gene causing thiamine transporter-2 deficiency can also result in WE [10]. Interestingly, a case report also described nonalcoholic WE secondary to a meat-only diet [11]. In addition, vomiting can cause thiamine deficiency, but it can also be a gastrointestinal manifestation of thiamine deficiency [12]. It is important to keep in mind that patients may present only with confusion, including disorientation and poor memory, and 19% of autopsy-diagnosed WE have none of the classical triad [13]. Therefore, reliance on classical triad can lead to underdiagnosis, and clinicians should maintain vigilance in the right clinical situation.

The typical MRI sites of FLAIR and T2 hyperintensity in WE include the thalami, mammillary bodies, tectum, and periaqueductal areas. The atypical sites of hyperintensity include the pons, cerebellar dentate nuclei, red nuclei, substantia nigra of the midbrain, cranial nerve nuclei, vermis and paravermian regions of the cerebellum, corpus callosum, fornices, head of the caudate nucleus, and frontal-parietal cortex [3]. Typical lesions are seen only in 58% of all cases of WE [14].

The most commonly used clinical criteria are the 2010 European Federation of Neurological Societies (EFNS) criteria, which have a diagnostic sensitivity of 85%. Using these criteria, the diagnosis of alcoholic WE requires two of the following: nutritional deficiency, oculomotor abnormalities, equilibrium disorders, and either an altered mental state or mild memory impairment. These criteria may be also used to diagnose WE in nonalcoholic patients [15]. Importantly, failure of oculomotor abnormalities to improve should alert to the possibility of another diagnosis. MRI has a sensitivity of only 53%, and a negative MRI should not preclude the diagnosis [14].

## Conclusions

Alcoholic and nonalcoholic WE differ in risk factors, clinical presentation, and even the extent of MRI findings. Nonalcoholic WE is likely an underdiagnosed clinical condition given the fact that patients are more likely to present with altered mental status and atypical MRI findings, and as such, these tend to have a worse prognosis. Clinical criteria have been developed to aid in the diagnosis of alcoholic WE but can be applied to nonalcoholic WE as well. Seizure is a rare but documented clinical presentation of WE. As this case demonstrates, early diagnosis and thiamine repletion can yield good control of seizures with eventual safe discontinuation of antiepileptic medications.
